# Eviscération transanale de l'intestin grêle chez l'enfant secondaire à une contusion de l'abdomen: à propos d'un cas

**Published:** 2011-12-02

**Authors:** Papa Abdoulaye Bâ, Sékou Amadou Soumah, Balla Diop, Mamadou Mour Traoré, Charfi Mahdi, Elhadji Malick Mbaye, Babacar Fall

**Affiliations:** 1 Service de Chirurgie Générale, Hôpital Régional de Thiès, Sénégal; 2 Service de Chirurgie Générale, Hôpital Saint Jean de Dieu de Thiès, Sénégal; 3 Service d'Anesthésie-réanimation, Hôpital Régional de Thiès, Sénégal; 4 Service de Chirurgie Générale, Hôpital Général de Grand Yoff Dakar, Sénégal

**Keywords:** Eviscération, intestin grêle, enfant, rectum, traumatisme, Sénégal

## Abstract

L’éviscération transanale de l'intestin grêle au cours d'un traumatisme fermé de l'abdomen est une situation exceptionnelle. Nous rapportons le cas d'une fille de 7 ans, reçue pour un état de choc hémorragique avec une éviscération transanale de l'intestin grêle suite à une contusion de l'abdomen. Ce tableau clinique est survenu au décours d'un accident de la circulation. La laparotomie exploratrice permettait de découvrir une plaie longitudinale de la face antérieure du rectum, située au dessus de la réflexion péritonéale, à travers laquelle passaient les anses grêles. Une suture de la plaie rectale associée à une iléostomie double après résection du grêle éviscéré et un à drainage du cul-de-sac de Douglas ont été réalisés. L'enfant est décédé en postopératoire précoce. A travers cette observation, les auteurs se proposent de revoir les cas précédemment décrits et de discuter les options thérapeutiques.

## Introduction

L’éviscération est plus notée dans les plaies que dans les contusions de l'abdomen. Ainsi, un traumatisme fermé de l'abdomen compliqué d'une éviscération transanale du grêle constitue un fait rare dans notre pratique quotidienne. Nous en rapportons un cas survenu chez un enfant dans un contexte de polytraumatisme. Cette observation est exceptionnelle en ce sens qu'elle constitue à notre connaissance le premier cas africain. Les auteurs se proposent de revoir les cas similaires rapportés dans la littérature et de discuter les principes du traitement.

## Observation

K. S, âgée de 07 ans, a été admise au service des urgences de l'hôpital régional de Thiès pour une issue des anses grêles à travers l'anus. Il s'agissait d'un piéton heurté par un véhicule. A l'examen clinique, la fille était dans un état de choc. Il existait une éviscération transanale de l'intestin grêle. L'examen de l'abdomen révélait une distension abdominale et une matité des flancs. Le reste de l'examen clinique permettait de noter un traumatisme fermé de l’épaule droite, un traumatisme fermé du coude droit et une fracture clinique du fémur gauche. Le bassin était cliniquement stable.

Après une réanimation, l'exploration chirurgicale par laparotomie médiane a été décidée. Elle permettait de découvrir un hémopéritoine significatif (1,5 litre), provenant d'une déchirure du mésentère. Les organes pleins ne présentaient pas de lésions. La tentative de réintégration de l′intestin grêle dans l′abdomen par une manœuvre de propulsion-traction était difficile. Nous avions procédé à une exérèse du grêle prolabé par voie abdominale sur environ 70 cm, suivie de son extraction par le bas. Cela nous a permis de découvrir une plaie longitudinale de la face antérieure du rectum mesurant 3 cm, située au dessus de la réflexion péritonéale. La cavité abdominale n’était pas souillée. Une suture de la plaie rectale associée à une iléostomie double et un à drainage du cul-de-sac de Douglas ont été réalisés. L'enfant est décédé deux heures de temps après la chirurgie dans un tableau de défaillance multiviscérale.

## Discussion

L’éviscération trans-anale du grêle est une situation spectaculaire et rare chez l'enfant. Seuls 20 cas pédiatriques ont été rapportés dans la littérature [1]. Ce phénomène est plus connu chez l'adulte où des formes spontanées ont été décrites, occasionnées par les facteurs d'hyperpression intra-abdominale comme les exonérations, la toux et les vomissements [2]. Le mécanisme étiologique est plutôt différent dans la population pédiatrique. En effet, la quasi-totalité des cas sont secondaires à un traumatisme sauf celui qui s'est produit après une quinte de toux chez un nourrisson de 4 mois [3]. Par ailleurs, la revue de littérature nous montre que seulement huit cas ont été rapportés chez l'enfant après un traumatisme fermé de l'abdomen (Tableau 1). D'autres mécanismes étiologiques traumatiques ont été rapportés chez l'enfant à savoir les traumatismes par empalement [4], les accidents d'aspiration sur bonde de piscine [2,5] et les abus sexuels [6].

**Tableau 1:** les cas similaires d’éviscération trans-anale de l'intestin grêle chez l'enfant secondaire à un traumatisme fermé de l'abdomen

observations	Auteurs	Mécanismes	Age	Sexe	Gestes	Stomie de protection	Suites
1	Corduk [1]	Contusion abdominale	2 ans	M	Résection du grêle Résection sigmoïdienne Réparation de la plaie rectale	non	Survie
2	Qureshi [7]	contusion abdominale	9 ans	M	Resection-anastomose	Non	Survie
3	Vesey [8]	contusion abdominale	7 ans	M	Résection du grêle Réparation plaie rectale	non	Survie
4	Ellul [9]	Contusion abdominale	14 ans	F	Resection partielle du rectum	non	Survie
5	Ellul [9].	Contusion abdominale	9 ans	M	Résection du grêle Réparation de la plaie rectale	Non	Survie
6	Rechner [10]	contusion abdominale	9 ans	M	Résection du grêle Réparation de la plaie rectale	oui	Survie
7	Roy [11]	Contusion abdominale	10 ans	M	Résection du grêle Réparation de la plaie rectale	oui	Survie
8	Quraishi [12]	Contusion abdominale	5 ans	M	Résection du grêle Réparation de la plaie rectale	oui	Survie
9	Notre cas	Contusion abdominale	7 ans	F	Résection du grêle Réparation plaie rectale	oui	Décès en postopératoire précoce

Les lésions associées (intra et/ou extra-abdominales) sont souvent sévères et peuvent rendre le tableau clinique plus dramatique [1,7–11].

**Figure 1:**
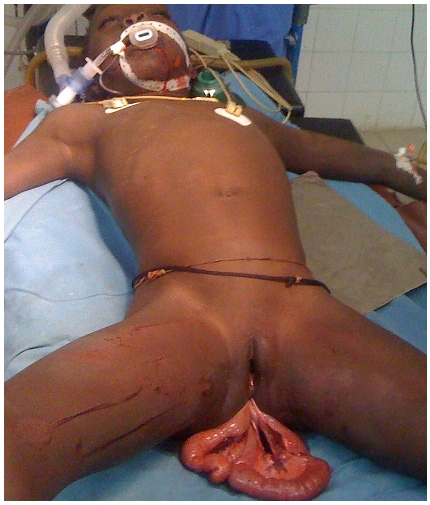
Anses grêles éviscérées à travers l'anus avec une déchirure du mésentère

La plaie rectale siège habituellement à la face antérieure du rectum prés de la réflexion péritonéale. En effet, cette zone est vulnérable à l'augmentation brutale de la pression intra-abdominale [3]. La réduction en douceur de l'intestin grêle éviscéré doit être de mise afin de limiter l’étendue de la résection intestinale. Quelque fois, la réintégration des anses éviscérées peut être difficile mais la dilatation de l'anus et l’élargissement de la plaie rectale pourraient faciliter cette réduction [5]. Tous ces gestes visent à préserver une bonne longueur du grêle dans le seul but d’éviter le syndrome de l'intestin court qui nécessite une nutrition parentérale à vie [10]. Bien souvent, la résection intestinale s'avère nécessaire en raison des lésions sévères sur le mésentère [1,4,10]. Ces lésions sont responsables d'une dévitalisation des anses éviscérées comme dans notre cas (Figure 1).

La fermeture primitive de la plaie rectale est indiquée si les lésions sont limitées et en l′absence de contamination fécale intra-péritonéale. Dans le cas contraire, la suture rectale est protégée par une colostomie de proche amont. Habituellement il n'existe pas de contamination fécale de la cavité péritonéale en raison du colmatage de la plaie rectale par les anses éviscérées [10,12]. Ce constat explique que certains préfèrent la fermeture de la plaie rectale sans dérivation des matières alors que d'autres optent pour une fermeture associée à une colostomie de proche amont (Tableau I). Pour notre part, nous avons choisi de faire une iléostomie pour protéger la suture rectale après la résection intestinale afin de limiter la durée de l'intervention. En dépit des lésions intra ou extra-abdominales sévères souvent associées, le pronostic est généralement bon, marqué par une survie dans tous les cas précédemment rapportés comme nous le montre le tableau 1. Le décès de notre patiente en postopératoire précoce dans un tableau de défaillance multiviscérale est lié à la précarité de nos conditions de travail malgré la promptitude de la prise en charge médico-chirurgicale. Ceci repose le débat sur l'insuffisance du plateau technique dans les hôpitaux éloignés de la capitale en Afrique subsaharienne.

## Conclusion

L’éviscération de l'intestin grêle par l'anus à travers une plaie rectale est rare chez l'enfant. Les formes survenues après un traumatisme fermé de l'abdomen sont exceptionnelles. Le traitement suit des principes de base bien définis.
